# Supplementary material for: Identification of photoperiod-induced specific miRNAs in the adrenal glands of Sunite sheep (Ovis aries)
Source: Front Vet Sci. 2022 Jul 22;9:888207. doi: 10.3389/fvets.2022.888207 (PMC9354845; doi:10.3389/fvets.2022.888207)
Supplement: Supplementary file 1 [file Data_Sheet_1.ZIP › Supplementary file/miRDeep2 file.zip!/miRDeep2 file/Novel_Structure/SP42A1/Novel_27.pdf]

Provisional ID	:	NC_019460.2_7182
Score total	:	1.4
Score for star read(s)	:	-1.3
Score for read counts	:	0
Score for mfe	:	1.1
Score for randfold	:	1.6
Score for cons. seed	:	
Total read count	:	33
Mature read count	:	33
Loop read count	:	0
Star read count	:	0

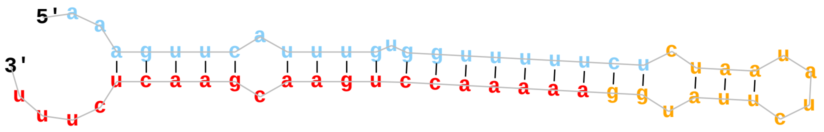

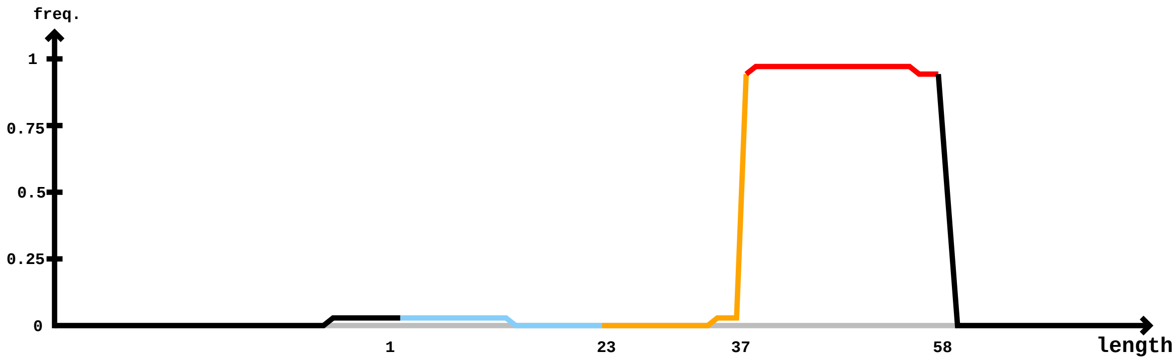

Star Mature

```
5'- agugcuaacuccacuguacuauuggcuuggcuaaaaaguucauuugugguuuuucucuaauaucuuauggaaaaaccugaacgaacucuuugggcagcccaaucauuugaaa   -3'    exp
    (((((..........)))))(((((.(((.(((((.(((((.((((.(((((((((.(((....))).))))))))))))).))))).))))).))).))))).........    reads       mm          sample
    ...........................uggcuGaaaaguucauuug..................................................................    1           1           seq
    ...................................................................uggaaaaaccugaaUgaacuc........................    1           1           seq
    ......................................................................aaaaaccugaacgaacuUuuu.....................    20          1           seq
    ......................................................................aaaaaccugaacgaacucuuug....................    1           0           seq
    ......................................................................aaaaaccugaacgaacuUuuug....................    11          1           seq
    .......................................................................aaaaccugaacgaacuUuuu.....................    1           1           seq
```
